# Liquid–liquid phase reaction between crystal violet and sodium hydroxide: kinetic study and precipitate analysis

**DOI:** 10.1098/rsos.220494

**Published:** 2022-10-12

**Authors:** Qingcheng Yu, Maya Florentino, Emily Abplanalp, Yingyi Liang, Sally Kremer, Gabriel Choi, Chris Park, Hye Jung Jung, Gary Halada, Steve Nitodas, Yizhi Meng, Taejin Kim

**Affiliations:** ^1^ Department of Materials Science and Chemical Engineering, Stony Brook University, Stony Brook, NY 11794, USA; ^2^ Ward Melville High School, East Setauket, NY 11733, USA; ^3^ Granada High School, Livermore, CA 94550, USA; ^4^ DaVinci College of General Education, Chung-Ang University, Seoul, South Korea

**Keywords:** crystal violet, kinetic parameter, UV-vis spectroscopy, Fourier transform infrared spectroscopy, solvent violet 9

## Abstract

To investigate reaction order and kinetic parameters of the reaction between crystal violet (CV) and sodium hydroxide (NaOH), various concentrations of the reactants were applied. The present work also verifies the unknown solid product produced under highly concentrated conditions. The reaction orders of CV and NaOH were determined to be 1 and 1.08 by pseudo rate method, respectively, with a rate constant, *k*, of 0.054 [(M^−1.08^) s^−1^]. In addition to pseudo rate method, the half-life approach was used to calculate the overall reaction order to verify the accuracy of pseudo rate method. The overall reaction order was determined to be 1.9 by the half-life method. The overall reaction order based on the two methods studied was approximately 2. The precipitate formation was observed when high concentrations of CV (0.01–0.1 M) and NaOH (1.0 M) were applied. Fourier transform infrared (FTIR) spectroscopy was used to compare the spectra of the precipitate generated and a commercial solvent violet 9 (SV9). Based on the FTIR spectra, it was confirmed that the molecular structure of the precipitate matched that of solvent violet 9.

## 1. Introduction

Crystal violet (CV, C_25_N_3_H_30_Cl) is a cationic triphenylmethane, which is used in biomedical fields, forensics and the textile industry as a strong dye chemical [1]. For example, in the popular Gram's method for identifying different types of bacteria, CV binds to the peptidoglycan layer of gram-positive bacteria, yielding its signature purple hue. By contrast, gram-negative bacteria lose this hue due to a thinner layer of peptidoglycan [2]. Additionally, CV is an integral part of a zinc carbonate CV stain that aids to detect fingerprints on non-porous surfaces [3]. Among the many applications, CV has been used extensively as a synthetic dye chemical. The molecular structure of CV contains alternating single and double bonds, which extend over three benzene rings and the central carbon atom. This extensive conjugation is the main cause of the coloured appearance of the dye [4].

Although the bleaching process is a widely used method to effectively remove dyes from fabric, bleach has long-term negative effects on both the human body and the environment and can damage the fabric such that it is no longer suitable for recycling [5,6]. These challenges are compounded by the fact that CV remains in the environment for long periods of time, and traditional methods (e.g. filtration, precipitation, adsorption and electrodialysis) for removing pollutants cannot be applied to CV due to its synthetic nature [7]. Not only does CV have the potential to contaminate the environment (e.g. soil and water), it also acts as a mitotic poison, clastogen and tumour growth promoter [8–10]. Additionally, a lifespan study by Littlefield *et al*. [11] showed an increase in both the prevalence of liver neoplasms and the rate of mortality with subjection to CV. With careful consideration of these detrimental effects, means of eliminating the potential threat of CV to both environmental and human health become exceedingly important.

In fundamental chemical reaction studies, sodium hydroxide (NaOH) has been applied due to its strong interaction with CV, which results in disruption of the extensive conjugation in the CV structure and colour removal. Since NaOH is readily available and easily stored, its reaction with CV is often used in educational contexts to study thermodynamic, kinetic and colorimetric data and to use various analytical tools (e.g. ultraviolet-visible (UV-vis) spectroscopy) [4]. Previously, Felix reported that CV decolorization with NaOH is an endothermic (ΔH = 13.95 kJ mol^−1^) and non-spontaneous (ΔG = 91.43 kJ mol^−1^) reaction [12]. Based on the abundance of literature regarding the kinetics of the reaction between CV and NaOH [13,14], the overall second-order reaction (first-order with respect to both CV and NaOH) is often considered, although there are a substantial number of studies where a precise reaction order for NaOH is calculated [4,13–16].

In this study, the CV and NaOH decolorization reaction was examined with varying concentrations of CV and NaOH. UV-vis spectroscopy was used to derive the rate equation and reaction order. To identify the final product, solvent violet 9 (SV9, C_25_H_31_N_3_O), formed at high concentrations of CV and NaOH, Fourier transform infrared (FTIR) spectroscopy was employed. It is worthwhile to note that most reported results do not show or discuss the presence or identification of precipitates. Based on our knowledge, this is the primary study analysing the precipitate and comparing it with commercial SV9. The methodology described herein could be applied for the conversion and removal of other natural and synthetic dye chemicals, especially from dyed fabrics.

## 2. Experimental section

## 2.1. Materials and sample preparation

Powdered anhydrous CV (C_25_N_3_H_30_Cl, CAS# 548-62-9, ACS reagent, greater than or equal to 90%) and NaOH pellets (NaOH, CAS# 1310-73-2, ACS reagent) were purchased from Sigma-Aldrich. Solvent violet 9 (SV9, C_25_H_31_N_3_O, CAS# 467-63-0, Tech grade) was purchased from BOC Sciences and used without further treatment. Deionized (DI) water (approx. 20 mΩ cm^−1^, Direct-Q3, Millipore Sigma) was used to make the CV and NaOH aqueous solutions. All samples were transferred using 100–1000 µl Reference 2 micropipettes (Eppendorf) with single-use standardization pipette tips (Fisherbrand). A 1.0 × 10^−2^ M CV stock solution was used to prepare the desired working solutions through serial dilution (i.e. 1.0 × 10^−3^, 1.0 × 10^−4^ and 1.0 × 10^−5^ M). For instance, to make a 500 ml volume of the 1.0 × 10^−3^ M solution, 50 ml of the 1.0 × 10^−2^ M CV solution was added to 450 ml of DI water. In the case of NaOH solutions, a 1.0 M NaOH stock solution was used to prepare the 0.05, 0.1, 0.3 and 0.5 M NaOH working solutions.

To collect the absorbance spectra, 50 µl of CV and 50 µl of NaOH solutions were pipetted into a microplate and placed into the UV-vis spectrometer. For the FTIR measurements, CV powder was mixed with a minimal amount of water to form a paste. If precipitates formed, the samples were separated from the solution and dried in an oven at 60°C for 48 h.

### 2.2. Characterization

**Scheme 1.**
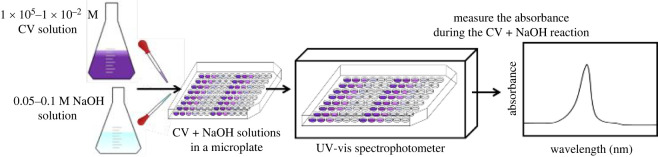
Schematics of the UV-vis spectroscopy measurement procedures.

The UV-vis spectra were obtained with a Tecan Infinite 200 PRO UV-visible spectrophotometer. The samples were pipetted (100–1000 µl Reference 2 micropipettes, Eppendorf) into the microplate (96-well Corning Falcon 351172 STERILE R), and measurements were conducted at room temperature (21°C) in the range of 400–700 nm with a step size of 5 nm and two flashes. The general UV-vis spectroscopy measurement procedures are shown in scheme 1.

To quantify changes in CV concentration during the reaction, an absorbance band at approximately 590 nm was monitored until the peak intensity was indistinguishable from the baseline reference point of 650–700 nm [17]. All absorbance data during the reaction was recorded using the i-control™ Microplate Reader Software 1.11 (for the Infinite reader). In addition to UV-vis spectroscopy, to discern the time for the reaction to reach completion, an extra batch of samples was prepared, and the colour change was recorded. The infrared (IR) spectra were obtained using a Nicolet iS50 FTIR spectrometer (Thermo Fisher Scientific) equipped with an added attenuated total reflectance accessory. The FTIR spectra were recorded in the range of 400–1800 cm^−1^ at a resolution of 4 cm^−1^ with 32 scans. A background spectrum was collected before each sample was analysed. The spectra were obtained using OMNIC software.

## 3. Results and discussion

### 3.1. Effect of crystal violet and sodium hydroxide concentration on crystal violet decolorization and precipitate formation

**Figure 1.**
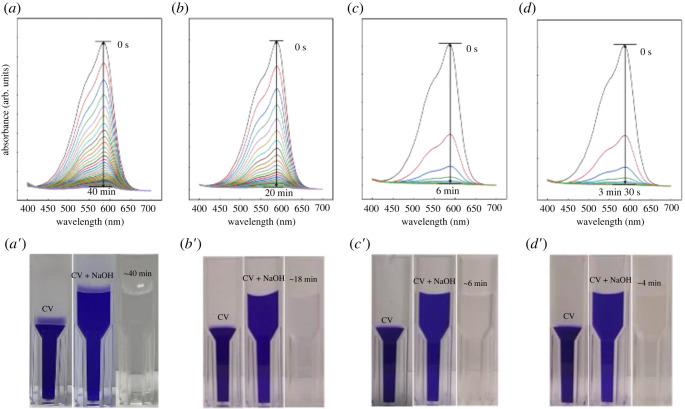
UV-visible spectra of reaction progression. (*a*) 1.0 × 10**^−^**^4^ M CV + 0.05 M NaOH, (*b*) 1.0 × 10**^−^**^4^ M CV + 0.1 M NaOH, (*c*) 1.0 × 10**^−^**^4^ M CV + 0.3 M NaOH and (*d*) 1.0 × 10**^−^**^4^ M CV + 0.5 M NaOH. Reaction conditions: temperature = 21°C, 100 µl total volume. Video capture of the reaction at CV solution only, initial CV reaction with NaOH and completed reaction. (*a*′) 1.0 × 10**^−^**^4^ M CV + 0.05 M NaOH, (*b*′) 1.0 × 10**^−^**^4^ M CV + 0.1 M NaOH, (*c*′) 1.0 × 10**^−^**^4^ M CV + 0.3 M NaOH and (*d*′) 1.0 × 10**^−^**^4^ M CV + 0.5 M NaOH. Reaction conditions: temperature = 21°C, total volume = 2 ml (1 : 1 ratio).

UV-vis spectroscopy was applied to analyse the CV concentration during the reaction with NaOH. As shown in figure 1, the maximum CV absorbance occurs at approximately 590 nm. As the concentration of NaOH was increased from 0.05 M to 0.5 M with a fixed concentration of CV (1.0 × 10^−4^ M), the reaction time decreased from 40 min to 3 min 30 s (figure 1*a–d*). Note that when a CV concentration of 1.0 × 10^−4^ M CV only was analysed (without NaOH), the absorbance peaks (500 nm–650 nm) showed extensive saturation, resulting in the absence of a clear peak (not shown for brevity). The obtained results were used to calculate the kinetic parameters. For comparison purposes, the video captured images are shown in figure 1*a*′–*d*′). As expected, the time needed for CV to decolorize decreased with increasing NaOH concentration.

**Figure 2.**
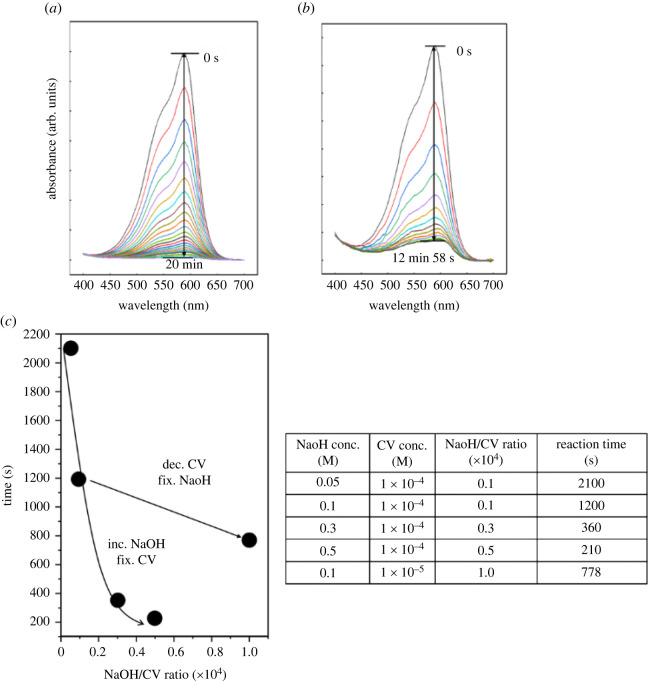
UV-visible spectra with intensity change over the progression of the CV/NaOH reaction. (*a*) 1.0 × 10**^−^**^4^ M CV + 0.1 M NaOH, (*b*) 1.0 × 10**^−^**^5^ M CV + 0.1 M NaOH and (c) NaOH/CV ratio effect on reaction time. Reaction conditions: temperature = 21°C, 100 µl total volume.

Figure 2 shows the UV-vis absorbance spectra with varied CV concentrations (1.0 × 10^−4^ M and 1.0 × 10^−5^ M), while the NaOH concentration was fixed at 0.1 M. As observed, the reaction time decreased from 20 min to 12 min 58 s with decreasing CV concentration. Although it was confirmed that decolorization efficiency can be improved with increasing NaOH concentration (figure 1*a*–*d*) or decreasing CV concentration (figure 2*a,b*), the reaction time was not fully dependent on the NaOH/CV concentration ratio (figure 2*c*). In the case of the 1.0 × 10^−5^ M CV and 0.1 M NaOH reaction (NaOH/CV ratio = 1.0 × 10^4^), the reaction time for CV decolorization was longer than that of the 1.0 × 10^−4^ M CV and 0.5 M NaOH reaction (NaOH/CV ratio = 0.5 × 10^4^). This result suggests that the efficiency of the CV decolorization reaction could be controlled by modulating the NaOH/CV ratio and the absolute value of the NaOH (or CV) concentrations. In the case of the 1.0 × 10^−5^ M CV and 0.1 M NaOH (NaOH/CV ratio = 1.0 × 10^4^) solution, it is also considered that the reaction time would have increased further because the CV and NaOH concentrations are low compared with 1.0 × 10^−4^ M CV and 0.3 M NaOH (NaOH/CV ratio = 0.3 × 10^4^) and 1.0 × 10^−4^ M CV and 0.5 M NaOH (NaOH/CV ratio = 0.5 × 10^4^). Because the 1.0 × 10^−5^ M CV and 0.1 M NaOH reaction (NaOH/CV ratio = 1.0 × 10^4^) showed a significantly shorter reaction time than that for 10^−4^ M CV and 0.1 M NaOH reaction (NaOH /CV ratio = 0.1 × 10^4^), based on the results shown in figures 1 and 2, it can be concluded that the absolute value of the NaOH concentration has more of an impact on the rate than the CV concentration.

### 3.2. Determination of reaction order and rate constant

CV is a relatively large molecule with three benzene rings and an amine bonded to a central carbon atom. Because NaOH is a strong base, it rapidly dissociates into Na^+^ and OH^−^ ions in solution, saturating the CV solution with hydroxide ions. A kinetic study of the decolorization of CV was performed based on the results presented in figures 1 and 2. The rate of the reaction is given by the generalized rate law:

3.1 $$\mathrm{Rate}=k{\left[{\mathrm{CV}}^{+}\right]}^{m}{\left[{\mathrm{OH}}^{-}\right]}^{n},$$

where *k* is the rate constant for the reaction, [CV^+^] and [OH^−^] express the concentrations of CV and hydroxide ions, respectively, *m* is the reaction order with respect to [CV^+^], and *n* is the reaction order with respect to [OH^−^].

With an excess of NaOH (i.e. NaOH/CV concentration ratio = 1.0 × 10^3^–1.0 × 10^4^), the rate equation could be simplified using the assumption that NaOH concentration can be considered constant throughout the reaction,

3.2 $$k^{\prime }=k{\left[{\mathrm{OH}}^{-}\right]}^{n}.$$

The rate constant was determined using a method involving a pseudo rate constant ($k^{\prime }$) to simplify the rate law,

3.3 $$\mathrm{Rate}=-\frac{d[{\mathrm{CV}}^{+}]}{dt}=k^{\prime }{\left[{\mathrm{CV}}^{+}\right]}^{m}.$$

The linear relationship between absorbance (A) and CV concentration is given by Beer's law,

3.4  A=εcl,

where ***ε*** is the molar absorption coefficient, ***c*** is the concentration and ***l*** is the optical path length.

To determine the reaction order (*m*), the CV concentration ([CV^+^], 0th order), the natural log of [CV^+^] (ln [CV^+^], 1st order) and the inverse of [CV^+^] (1/[CV^+^], 2nd order) were plotted as a function of reaction time based on the integrated rate law,

3.5 $$0\mathrm{th}\;\mathrm{order}:\;-\frac{d[{\mathrm{CV}}^{+}]}{{\left[{\mathrm{CV}}^{+}\right]}^{0}}=k^{\prime }dt\;\to [{\mathrm{CV}}^{+}]=-k^{\prime }t,$$

3.6 $$1\mathrm{st}\;\mathrm{order}:\;-\frac{d[{\mathrm{CV}}^{+}]}{{\left[{\mathrm{CV}}^{+}\right]}^{1}}=k^{\prime }dt\;\to \ln[{\mathrm{CV}}^{+}]=\ln{\left[{\mathrm{CV}}^{+}\right]}_{0}-k^{\prime }t$$

3.7 $$\mathrm{and}\;2\mathrm{nd}\;\mathrm{order}:\;-\frac{d[{\mathrm{CV}}^{+}]}{{\left[{\mathrm{CV}}^{+}\right]}^{2}}=k^{\prime }dt\;\to \;\frac{1}{\left[{\mathrm{CV}}^{+}\right]}=\frac{1}{{\left[{\mathrm{CV}}^{+}\right]}_{0}}+k^{\prime }t.$$

**Figure 3.**
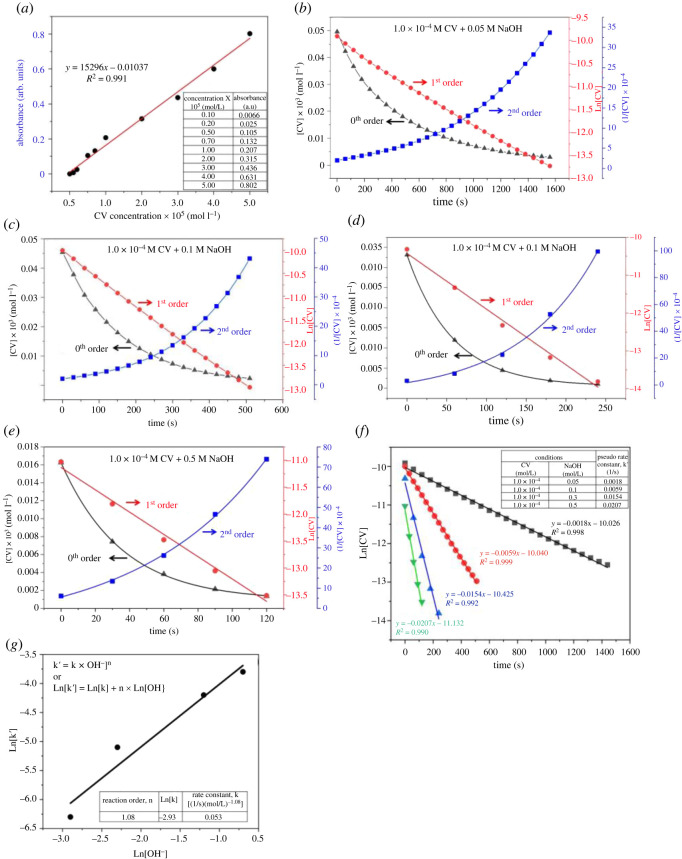
(*a*) Calibration plot for UV-vis absorbance versus CV concentration. Determination of the reaction order with respect to CV (*b*) 1.0 × 10**^−^**^4^ M CV + 0.05 M NaOH, (*c*) 1.0 × 10**^−^**^4^ M CV + 0.1 M NaOH, (*d*) 1.0 × 10**^−^**^4^ M CV + 0.3 M NaOH and (*e*) 1.0 × 10**^−^**^4^ M CV + 0.5 M NaOH. (*f*) Natural log of [CV] over time for three trials at varying hydroxide concentrations, fixed CV concentration: 0.0001 M. (*g*) ln [*k*′] versus ln [OH^−^] graph for hydrolysis of CV at 1.0 × 10**^−^**^4^ M CV and varying NaOH concentrations.

As shown in figure 3*b–e*, all datasets are linear when the natural log of [CV^+^] is plotted against reaction time, indicating that the order dependence with respect to [CV^+^] is 1st order, which is in accordance with previous studies [4,12,13,18].

While the conclusion of first-order kinetics with respect to [CV^+^] coincides with previous literature findings, the precise values of *k* and *n* were calculated to further investigate the reaction kinetics. According to equation (3.6), the pseudo rate constant, *k′*, is determined by the slope of the linearized graphs found using the absorbance data. Obtaining pseudo rate constants for different concentrations of hydroxide allows a system of linear equations to be solved. Reaction order, *n*, with respect to [OH^−^] and the reaction rate constant, *k*, are based on two solutions in this system.

Taking the natural logarithm of both sides of equation (3.2), the following equation is obtained:

3.8 $$\ln(k^{\prime })\;=n*(\ln[{\mathrm{OH}}^{-}]\;)+\ln(k).$$

Regression analysis on a plot of ln([*k*′]) against ln([OH^−^]) can be readily carried out to determine *n* and *k*. This approach improves accuracy by incorporating data from all three runs, rather than using only two runs, as reported in previous studies [15,16]. Therefore, when ln([*k*′]) is plotted against ln([OH^−^]), the slope will be linear. This slope provides the reaction order (*n*) with respect to [OH^−^] and the *y*-intercept represents the *k* value.

As shown in figure 3*f*, the plots of ln([CV+]) against time are linear and the slope is the pseudo rate constant *k′*. In addition, figure 3*g* shows the plot of ln([*k′*]) against ln([OH^−^]) for the various NaOH concentrations and derived *k′* values. It is observed that the plot yields a straight line, which is consistent with the derivation of equation (3.8). Based on figure 3*g*, the rate order with respect to [OH^−^], *n*, was determined as 1.08 and the reaction rate constant, *k*, was determined as 0.054 (M^−1.08^) s^−1^. This quantitative rate law allows the kinetics of the equation to be understood, as it is apparent from the reaction order that changing the amount of hydroxide affects the reaction rate more than changing the amount of CV would. These results are supported qualitatively as well, given that the hydroxide functional group is smaller than the CV molecule. More hydroxide in solution would lead to more frequent intermolecular collisions.

The obtained reaction order and rate constants were compared with previous studies, and the results are displayed in table 1.

**Table 1.** Comparison of rate constants and reaction orders.

experimental conditions			rate constant (*k*)	*m*	*n*	reference
temp. (°C)	CV conc. (M)	NaOH conc. (M)				
6°C	2.600 × 10^−6^	0.008–0.024	0.032 [s^−1^]	0.38	0.62	[4]
21°C	2.600 × 10^−6^	0.008–0.024	0.120 [s^−1^]	0.24	0.76	[4]
30°C	8.812 × 10^−5^	0.04	0.130 [M^−1^ s^−1^]	1.00	1.00	[13]
7.5°C	1.985 × 10^−5^	0.01–0.05	0.034 [M^−1^ s^−1^]	1.00	1.00	[14]
21°C	1.985 × 10^−5^	0.01–0.05	0.118 [M^−1^ s^−1^]	1.00	1.00	[14]
45°C	1.985 × 10^−5^	0.01–0.05	0.721 [M^−1^ s^−1^]	1.00	1.00	[14]
room temp.^a^	1.0 × 10^−5^	0.004–0.008	0.160 [M^−1^ s^−1^]	1.00	1.00	[15]
room temp.^a^	2.000 × 10^−5^	0.02–0.10	0.170 [M^−1^^.^^06^ s^−1^]	1.00	1.06	[16]
21°C	1.0 × 10^−5^ –1.0 × 10^−4^	0.1–0.50	0.042 [M^−0^^.^^85^ s^−1^]	1.00	0.85	current research

^a^The value of reaction temperature was not reported.

Many previous studies determined that the reaction order in respect to both CV and NaOH is first order, which is well matched to the current results, while the obtained [OH^−^] reaction order (1.08) was slightly higher than the reported value. The obtained rate constant, however, is quite different compared with the literature data, even at similar reaction temperatures. It is assumed that higher CV and NaOH concentrations under the current experimental conditions could contribute to the lower rate constant. For instance, most previous literature indicated that the concentration of NaOH fell between 0.0001 and 0.09 M [4,13–16], while the current hydrolysis reaction was carried out at higher (0.1–0.5 M) NaOH concentrations. Highly concentrated solutions of NaOH may lead to a difference in the reaction rate constant, which requires further investigation. Based on the literature results, it is concluded that several reaction conditions such as mixing conditions, concentrations of reactants (CV or NaOH) and reaction temperatures, could contribute to the discrepancy of rate constants [4,13–16]. The steady state or transient state condition may also affect the rate constant values. Thompson & Jason [14] reported that the first 30 s of data were ignored when calculating the reaction orders and rate constant, as the solutions were mixing in the cuvette during this time. Another variation in the rate constants may arise due to the selection of different CV peak wavelengths (i.e. 530, 590 and 595 nm) from the UV-vis spectra, although this effect is expected to be minor [13,14]. As shown in table 1, it is clear that the reaction rate is directly related to the reaction temperature. For instance, Salahudeen & Rasheed and Thompson & Jason studied the effects of temperature on CV decomposition and reported that the rate constant increased with increasing reaction temperature [4,14].

### 3.3. Overall reaction order from half-life method

Considering the error caused by the pseudo rate method, which assumes that the reaction order with respect to CV is 1, the half-life method was employed to calculate the overall reaction order of the hydrolysis of CV. This method assumes that the consumption of NaOH is proportional to the consumption of CV. Therefore, the consumption ratio (*c*) for the reactants is constant.

3.9 $$\frac{\left[{\mathrm{OH}}^{-}\right]}{\left[{\mathrm{CV}}^{+}\right]}=c.$$

Thus, the hydrolysis reaction in equation (3.1) can be written as follows:

3.10 $$\mathrm{Rate}=-\frac{d[CV^{+}]}{dt}=\hat{k}{\left[{\mathrm{CV}}^{+}\right]}^{x},$$

where $\hat{k}=k\cdot c$ and x=m+n (overall reaction order).

The following equation (3.11) represents the relationship between $\left[{\mathrm{CV}}^{+}\right]$ and reaction time (*t*):

3.11 $${\left[{\mathrm{CV}}^{+}\right]}_{t}^{1-x}-{\left[{\mathrm{CV}}^{+}\right]}_{0}^{1-x}=\hat{k}\;(x-1)\;t.$$

Since the half-life of the reaction, $t_{1/2}$, is defined as the time required for the reactant concentration to fall to half of its original value, equation (3.11) can be re-written as equation (3.12) and equation (3.13),

3.12 $$t_{1/2}=\frac{{\left(0.5\right)}^{1-x}-1}{\hat{k}\;(x-1)}{\left[{\mathrm{CV}}^{+}\right]}_{0}^{1-x}$$

and

3.13 $$\ln t_{1/2}=(1-x)\;\ln{\left[{\mathrm{CV}}^{+}\right]}_{0}+\ln\left(\frac{0.5^{1-x}-1}{\hat{k}\;(x-1)}\right).$$

**Figure 4.**
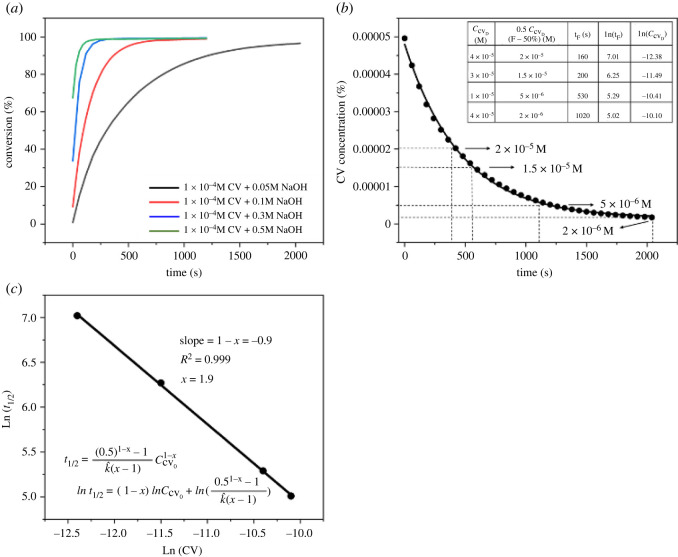
(*a*) Conversion factor versus time for different concentrations of NaOH. (*b*) CV concentration versus time for 1.0 × 10**^−^**^4^ M CV + 0.05 M NaOH sample. (*c*) $\ln t_{1/2}$ versus $\ln{\left[CV^{+}\right]}_{0}$ for the various CV initial concentrations.

Regression analysis on a plot of $\ln\;t_{1/2}$ against $\ln{\left[{\mathrm{CV}}^{+}\right]}_{0}$ can be readily performed to determine the overall reaction order *x* [19]. Figure 4*a* shows the CV conversion as a function of reaction time for different concentrations of NaOH. Considering the rapid reaction at the beginning of the hydrolysis, the sample with the lowest NaOH initial concentration, 0.05 M, was chosen for further analysis. As shown in figure 4*b*, the CV concentration decreased exponentially with increasing reaction time. Four points were chosen to calculate the overall reaction order (*x*). Figure 4*c* depicts the plot of $\ln t_{1/2}$ and $\ln{\left[{\mathrm{CV}}^{+}\right]}_{0}$ for the various CV initial concentrations and the half-life derived from figure 4*b*. Based on figure 4*c*, the overall reaction order was determined to be 1.90 by using the half-life approach. Although this value is slightly lower than that of the pseudo rate constant method, 2.17, the overall reaction order of the CV and NaOH reaction can be estimated to be approximately 2 at room temperature (21°C). This result is consistent with previous studies as shown in table 1.

### 3.4. Analysis of precipitate chemical

**Figure 5.**
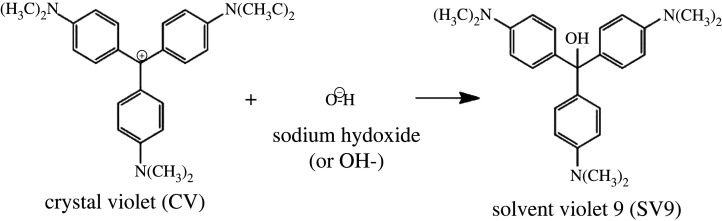
Solvent violet 9 formation through the CV and NaOH reaction.

Although the reaction times varied with different CV and NaOH concentrations (figures 1 and 2), due to the excess of NaOH, most CV molecules were completely converted into a new compound, solvent violet 9 (SV9), as shown in figure 5 [18].

**Figure 6.**
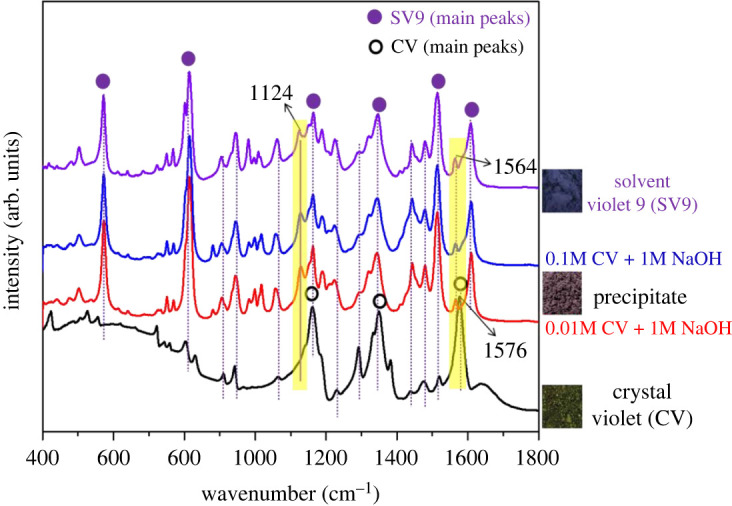
FTIR spectra in the range of 400–1800 cm^−1^ for solvent violet 9 (SV9), solid CV paste, precipitate of 0.1 M CV + 1 M NaOH (reaction time: 1 hr and 30 min) and precipitate of 0.01 M CV + 1 M NaOH (reaction time: 3 min). Reaction conditions: temperature = 21°C, total volume = 20 ml (1 : 1 ratio), stirred at 150 r.p.m. in 250 ml beaker. The precipitate was collected through filtration and drying at 60°C for 48 h.

To confirm the existence of SV9, high concentrations of CV (i.e. 0.1 M and 0.01 M) and 1.0 M NaOH were applied. It should be noted that the precipitate had an intense dark colour (photo in figure 6)), in contrast with the colourless solution (figure 1*a*′–*d*′)) produced when the reactants were lower in concentration. This phenomenon is probably due to the non-spontaneous nature of the high-concentration reaction, as it did not proceed to completion with an excess of CV to decolorize. To further study the molecular structure of these precipitates, FTIR spectroscopy was used. For comparison purposes, solid CV and solid SV9 samples were analysed, with the results displayed in figure 6. The spectrum representing the untreated, solid CV contains strong peaks at approximately 1162 cm^−1^, approximately 1349 cm^−1^ and approximately 1576 cm^−1^, which correspond to C-N stretching vibration (or C-H stretching in aromatic ring), C-N stretching of aromatic tertiary amine (or C-H deformation in methyl group) and C=C stretching of the benzene ring, respectively [20–23]. The spectra of the precipitates show that most peak positions are very similar to those found in the spectrum for solid CV, while the intensity of the peaks changed drastically. For instance, the intensity of the approximately 1576 cm^−1^ peak in the CV spectrum decreased after the reaction, and the peak shifted to approximately 1564 cm^−1^. Upon closer inspection, the precipitate spectra were found to contain a weak peak at approximately 1124 cm^−1^ corresponding to C-O stretching in the tertiary alcohol, which is not present in the CV structure [24]. Compared with the spectrum of SV9, the spectra of the precipitates clearly show that the peak positions and intensities are well matched. Therefore, it is reasonable to conclude that the product formed after CV reacted with NaOH is SV9.

## 5. Conclusion

In this work, the reaction between CV and NaOH was investigated using UV-visible spectroscopy, FTIR spectroscopy and video imaging. UV-vis spectroscopy was used for quantitative analysis, specifically for the reaction order and rate constant derivation, while video imaging was used for qualitative analysis. The reaction orders of CV and NaOH are 1.00 and 1.08, respectively, and the calculated rate constant (*k*) is 0.054 [(M^−1.08^) s^−1^]. Another method using the half-life approach determined the overall reaction order to be 1.9. Because the results differ only slightly, the overall reaction order of the CV and NaOH reaction can be estimated to approximately 2 at room temperature (21°C), which matches previous studies. FTIR spectroscopy was used to study the molecular structure and bonding vibration of CV, the precipitate, and SV9. When high concentrations of both NaOH and CV reacted, a precipitate formed, which was concluded to be SV9 by FTIR analysis.

## Data Availability

All relevant necessary data to reproduce all results in the paper are within the main text and the Dryad Digital Repository: https://doi.org/10.5061/dryad [25].

## References

[1] Adak A, Bandyopadhyay M, Pal A. 2005 Removal of crystal violet dye from wastewater by surfactant-modified alumina. Sep. Purif. Technol. **44**, 139-144. (10.1016/j.seppur.2005.01.002)

[2] Rohatgi R, Sodhi G, Kapoor A. 2015 Small particle reagent based on crystal violet dye for developing latent fingerprints on non-porous wet surfaces. Egypt. J. Forensic Sci. **5**, 162-165. (10.1016/j.ejfs.2014.08.005)

[3] Sodhi G, Kaur J. 2012 A novel fluorescent small particle reagent for detecting latent fingerprints on wet non-porous items. Egypt. J. Forensic Sci. **2**, 45-47. (10.1016/j.ejfs.2012.04.004)

[4] Salahudeen N, Rasheed A. 2020 Kinetics and thermodynamics of hydrolysis of crystal violet at ambient and below ambient temperatures. Sci. Rep. **10**, 1-9. (10.1038/s41598-020-78937-4) 33318585 PMC7736580

[5] Markandeya S, Shukla SP, Mohan D. 2017 Toxicity of disperse dyes and its removal from wastewater using various adsorbents: a review. Res. J. Environ. Toxicol. **11**, 72-89. (10.3923/rjet.2017.72.89)

[6] Toren K, Blanc P. 1997 The history of pulp and paper bleaching: respiratory-health effects. Lancet **349**, 1316-1318. (10.1016/S0140-6736(96)10141-0) 9142078

[7] Ganea I, Nan A, Baciu C, Turcu R. 2021 Effective removal of crystal violet dye using neoteric magnetic nanostructures based on functionalized poly(benzofuran-co-arylacetic acid): investigation of the adsorption behaviour and reusability. Nanomaterials **11**, 1-15. (10.3390/nano11030679) PMC7999123 33803300

[8] Mani S, Bharagava R. 2016 Exposure to crystal violet, its toxic, genotoxic and carcinogenic effects on environment and its degradation and detoxification for environmental safety. Rev. Environ. Contam. Toxicol. **237**, 71-104. (10.1007/978-3-319-23573-8_4) 26613989

[9] Au W, Pathak S, Collie C, Hsu T. 1978 Cytogenetic toxicity of gentian violet and crystal violet on mammalian cells in vitro. Mutat. Res. Genet. Toxicol. Environ. Mutagen **58**, 269-276. (10.1016/0165-1218(78)90019-8) 745616

[10] Fan H, Huang S, Chung W, Jan J, Lin W, Chen C. 2009 Degradation pathways of crystal violet by Fenton and Fenton-like systems: condition optimization and intermediate separation and identification. J. Hazard. Mat. **171**, 1032-1044. (10.1016/j.jhazmat.2009.06.117) 19604632

[11] Littlefield N, Blackwell B, Hewitt C, Gaylor D. 1985 Chronic toxicity and carcinogenicity studies of gentian violet in mice. Fund. Appl. Toxicol. **5**, 902-912. (10.1016/0272-0590(85)90172-1) 4065463

[12] Felix L. 2018 Kinetic study of the discoloration of crystal violet dye in sodium hydroxide medium. J. Chem. Appl. Chem. Eng. **2**, 1-4.

[13] Potrich E, Amaral L. 2017 Determination of kinetic parameters of the crystal violet reaction with sodium hydroxide applying absorbance technique and the laws of Lambeert-Beer and Arrhenius. Enciclopédia Biosfera **14**, 1852-1861. (10.18677/EnciBio_2017A153)

[14] Thompson J, Jason T. 2004 A simple, inexpensive water-jacketed cuvette for the Spectronic 20. Chem. Educ. **81**, 1341-1343. (10.1021/ed081p1341)

[15] Corsaro G. 1964 Colorimetric chemical kinetics experiment. J. Chem. Educ. **41**, 48-50. (10.1021/ed041p48)

[16] Kazmierczak N, Griend D. 2017 Improving student results in the crystal violet chemical kinetics experiment. J. Chem. Educ. **94**, 61-66. (10.1021/acs.jchemed.6b00408)

[17] Muthuchudarkodi R. 2016 Synthesis, characterization of zirconia doped and undoped CdSe nanoparticles for photocatalytic degradation of organic dyes. EJSOC **1**, 1-8.

[18] Knutson T, Knutson C, Mozzetti A, Campos A, Haynes C, Penn R. 2015 A fresh look at the crystal violet lab with handheld camera colorimetry. J. Chem. Educ. **92**, 1692-1695. (10.1021/ed500876y)

[19] Levenspiel O. 1999 Chemical reaction engineering, 3rd edn., vol. 668, pp. 48-62. New York, NY: John Wiley & Sons, Inc.

[20] Alyami A, Barton K, Lewis L, Mirabile A, Iacopino D. 2019 Identification of dye content in colored BIC ballpoint pen inks by Raman spectroscopy and surface-enhanced Raman scattering. J. Raman Spectrosc. **50**, 115-126. (10.1002/jrs.5512)

[21] Abdi M, Balagabri M, Karimi H, Hossini H, Rastegar S. 2020 Degradation of crystal violet (CV) from aqueous solutions using ozone, peroxone, electroperoxone, and electrolysis processes: a comparison study. Appl. Water Sci. **10**, 1-10. (10.1007/s13201-020-01252-w)

[22] Cheriaa J, Khaireddine M, Rouabhia M, Bakhrouf A. 2012 Removal of triphenylmethane dyes by bacterial consortium. Sci. World J. **2012** 1-9. (10.1100/2012/512454) PMC3353484 22623907

[23] Kant A, Gaijon P, Nadeem U. 2013 Adsorption equilibrium and kinetics of crystal violet dye from aqueous. Chem. Sci. Rev. Lett. **3**, 1-13.

[24] Alavi M, Dehestaniathar S, Mohammadi S, Maleki A, Karimi N. 2020 Antibacterial activities of phytofabricated ZnO and CuO NPs by *Mentha pulegium* leaf/flower mixture extract against antibiotic resistant bacteria. Adv. Pharm. Bull. **11**, 497-504. (10.34172/apb.2021.057) 34513624 PMC8421631

[25] Kim T. et al. 2022 Data from: Liquid-liquid phase reaction between crystal violet and sodium hydroxide: kinetic study and precipitate analysis. *Dryad Digital Repository.* (10.5061/dryad.5dv41ns7v) PMC9554514 36312564

